# One artery to rule them all: A case of single coronary artery arising from the right sinus of Valsalva

**DOI:** 10.1016/j.radcr.2024.10.005

**Published:** 2024-10-30

**Authors:** Nikolaos S. Ioakeimidis, Panagiotis Pepis, Konstantina Mitrousi, Dimitrios Valasiadis

**Affiliations:** a General Hospital of Florina “Eleni Th. Dimitriou”, Department of Cardiology, Egnatias 9, Florina 53100, Greece; b Department of Cardiothoracic Surgery, AHEPA University Hospital, Aristotle University of Thessaloniki, Kiriakidi 1, Thessaloniki 54636, Greece; c Diagnostic Center “Ippokrateio - Ygeia Ptolemaidas”, Department of Cardiac Imaging, Trapezountos 44, Ptolemaida 50200, Greece

**Keywords:** Coronary vessel anomalies, Coronary arteries, CT angiography, Coronary angiography

## Abstract

A single coronary artery (SCA) is a rare congenital anomaly with an incidence of 0.024 - 0.066% in angiographies and potential implications for adverse events depending on the course of the anomalous artery. We present a unique case of a single coronary artery arising from the right sinus of Valsalva. A 77-year-old female presented to the emergency department with an ongoing 3-hour episode of palpitations and intermittent atypical chest pain over 1 week. Her medical history included hypertension and dyslipidemia. Electrocardiography revealed atrial fibrillation with rapid ventricular response, which was successfully managed with intravenous amiodarone and the diagnostic workup ruled out life threatening thoracic pathology including cardiac ischemia. A CT coronary angiography was performed due to the moderate pretest probability of coronary artery disease. The scan identified a single coronary artery with a common origin of the left main coronary artery (LMCA) and the right coronary artery (RCA), from the right sinus of Valsalva, classified as Lipton Type RII-A which is a benign variant. This case highlights the importance of identifying CAAs, which are often incidental but may have clinical relevance depending on the anatomical course and associated risk factors. Early and accurate diagnosis through advanced imaging techniques is crucial to guide appropriate management and ensure optimal outcomes for the patient.

## Introduction

Coronary artery anomalies (CAAs) represent a diverse group of congenital disorders, characterized by deviations in the origin, course, or structure of the coronary arteries and exhibit an incidence of 5.64% on coronary angiography [1]. CAAs have drawn increasing clinical attention due to their potential association with adverse cardiovascular events, including myocardial ischemia, arrhythmias, and sudden cardiac death, particularly in young athletes and otherwise healthy individuals [2,3].

The vast majority of CAAs are asymptomatic and often discovered incidentally during coronary angiography or advanced imaging modalities such as computed tomography coronary angiography (CTCA) and cardiac magnetic resonance (CMR) [4,5]. However, specific variants, such as anomalous coronary arteries with an inter-arterial course (between the aorta and pulmonary artery), may pose a significant risk due to the possibility of external compression during physical exertion or heightened stress [6].

Understanding the clinical significance of CAAs is crucial for both accurate diagnosis and appropriate management. In this case report, we present a unique case of a single coronary artery arising from the right sinus of Valsalva, highlighting the diagnostic process, clinical implications and management considerations for this rare anomaly. This case contributes to the limited body of literature on SCAs and underscores the importance of individualized risk assessment and treatment in patients with such coronary anomalies.

## Case report

A 77-year-old female presented to the emergency department of our secondary care hospital with a 3-hour complaint of ongoing palpitations and intermittent episodes of atypical chest pain over the past week. Her medical history was notable for well-controlled arterial hypertension and dyslipidemia. Upon arrival, an electrocardiogram (ECG) demonstrated atrial fibrillation with rapid ventricular response. Transthoracic echocardiography revealed no significant structural abnormalities, including normal left ventricular function and no evidence of valvular pathology.

The patient was treated with intravenous titrated amiodarone, which successfully restored an ectopic atrial rhythm, consistent with her prior ECG findings over the past 2 years. Serum cardiac enzymes, including troponin, were negative for myocardial ischemia. After cardioversion, the patient was clinically stable and she was discharged with instructions to initiate a direct oral anticoagulant (DOAC) for stroke prevention as well as a beta-blocker for rate control.

Given the patient's complaint of atypical chest pain and her moderate cardiovascular risk, further evaluation for coronary artery disease was pursued. The clinical pretest probability for significant coronary artery disease (CAD), calculated using the CAD Consortium model, was estimated at 20%, indicating the option of a noninvasive coronary evaluation. Accordingly, a CTCA was performed.

**Fig. 1:**
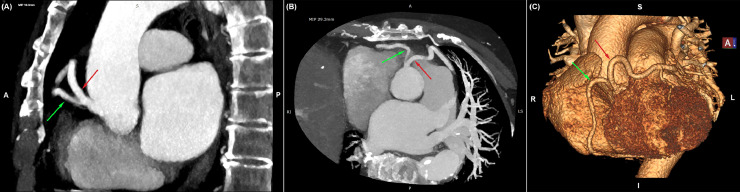
*(A)* and *(B)* Sagittal and axial oblique maximum intensity projection (MIP) CTCA images revealing a single ostium in the right sinus of Valsalva giving rise to the left main coronary artery (LMCA - red arrow) and the right coronary artery (RCA -green arrow) *(C)* 3D volume rendered image visualizing the anomalous common origin of the LMCA (red arrow) and RCA (green arrow). The profoundly long LMCA follows a course anterior to the RVOT and then splits to the left anterior descending artery and the circumflex artery.

CTCA revealed a calcium score of 2 Agatston Units (AU), indicating minimal coronary calcification. A small area of nonobstructive coronary artery disease (CAD-RADS 1) was identified in the distal left main coronary artery (LMCA). However, an unexpected finding was the presence of a coronary artery anomaly: both the LMCA and right coronary artery (RCA) originated from a single ostium in the right sinus of Valsalva (Figs. 1A and B). The LMCA followed a long course anterior to the right ventricular outflow tract (RVOT) before bifurcating into the left anterior descending (LAD) and left circumflex (LCx) arteries (Fig. 1C). The RCA exhibited a normal anatomical course. Based on these findings, the coronary anomaly was classified as a Lipton Type RII-A single coronary artery anomaly [7].

Given the absence of typical ischemic symptoms or significant obstructive CAD, conservative management was recommended, with a plan for continued medical therapy and routine follow-up.

## Discussion

A single coronary artery, defined as a sole coronary artery arising from the aortic trunk by a single ostium, is a rare congenital anomaly with an incidence of 0.024%-0.066% in angiographic cases [8]. These anomalies are further classified using the Lipton system, and in this case, the anomaly was classified as Lipton Type RII-A, where both the left main coronary artery (LMCA) and right coronary artery (RCA) share a common origin from the right sinus of Valsalva and the LMCA courses anterior to the right ventricular outflow tract (RVOT) [7] (Table 1).

**Table 1:** Lipton's classification of isolated single coronary artery types with additional descriptions from Yamanaka et al. [1,7].

Features	Coding and description
**Location of SCA ostium**	**L**: **L**eft sinus of Valsalva
	**R**: **R**ight sinus of Valsalva
**Branching pattern (anatomical distribution)**	**I**: the SCA courses as expected for a normal left (**L I**) or right (**R I**) coronary artery. In each case the contralateral area is supplied by long terminal branches of the SCA.
	**II**: after arising from the single ostium in the right (**R II**) or left (**L II**) aortic sinus, a large transverse trunk arising from the proximal part of the SCA crosses the base of the heart and supplies the contralateral area (the area where a normal coronary artery would be expected)
	**III**: describes the case of a SCA exclusively from the right coronary sinus with the LCx and LAD arising separately from a common trunk at the proximal part of the artery (**R III**)
**Course of the transverse trunk**	**A**: **A**nterior to the large vessels (anterior to the right ventricle)
	**B**: **B**etween the aorta and pulmonary artery
	**P**: **P**osterior to the large vessels
	*S: **S**eptal type in which part of the trunk courses through the interventricular septum
	*C: **C**ombined type (variety of different courses)

LAD, Left Anterior Descending artery; LCx, Left Circumflex artery; SCA, Single Coronary Artery.

⁎ Course descriptions added by Yamanaka & Hobbs (1990) to the original Lipton's classification [1].

In the presented case, the patient's symptoms were likely due to atrial fibrillation rather than ischemia, as cardiac enzymes were negative and the coronary anatomy did not demonstrate significant external compression or flow limitation. This is supported by the nonobstructive findings in the distal LMCA, and the patient's coronary calcium score of 2, indicating minimal atherosclerotic burden.

CT Coronary Angiography has become a crucial tool for the noninvasive diagnosis of CAAs. In a retrospective study of 2,572 patients who underwent CTCA the total prevalence of CAAs was 2.33% and the prevalence of SCA was 0.12% [9]. CTCA allows for precise and high quality anatomical visualization and risk stratification in cases where coronary anomalies are suspected or found incidentally [10]. In our case, CTCA provided a detailed understanding of the coronary anatomy, ruling out high-risk inter-arterial courses or other potential obstructive features. The patient's coronary anomaly was noted to have a benign course, and given the absence of symptoms directly attributable to the anomaly, conservative management was deemed appropriate.

Management strategies for CAAs vary depending on the type of anomaly, associated symptoms, and the risk of ischemic complications [11]. For patients with benign CAAs and no evidence of myocardial ischemia, conservative management with medical therapy and routine follow-up is often sufficient. In contrast, surgical intervention is recommended in high-risk cases, particularly when the anomalous coronary artery courses between the aorta and pulmonary artery, posing a risk for external compression during periods of increased cardiac output [12].

This case highlights the importance of recognizing coronary artery anomalies, even when discovered incidentally, as part of a thorough cardiovascular evaluation. While CAAs are rare, understanding their classification, potential complications, and appropriate management is crucial to prevent adverse outcomes. Clinicians should remain vigilant in using advanced imaging techniques such as CTCA, which not only provide diagnostic clarity but also guide the management of such anomalies based on the patient's risk profile.

In conclusion, this case of a Lipton Type RII-A single coronary artery anomaly demonstrates that in the absence of obstructive disease or ischemia, conservative management is a reasonable approach. Continued research and case reports are essential to further define the long-term prognosis and management strategies for patients with rare coronary artery anomalies like SCAs.

## Patient consent

The patient provided written informed consent regarding this publication.
